# Phosphorylated and Nonphosphorylated PfMAP2 Are Localized in the Nucleus, Dependent on the Stage of* Plasmodium falciparum* Asexual Maturation

**DOI:** 10.1155/2016/1645097

**Published:** 2016-07-25

**Authors:** Farah Aida Dahalan, Hasidah Mohd Sidek, Mogana Das Murtey, Mohammed Noor Embi, Jamaiah Ibrahim, Lim Fei Tieng, Nurul Aiezzah Zakaria, Noraishah Mydin Abdul-Aziz

**Affiliations:** ^1^School of Biosciences & Biotechnology, Faculty of Science and Technology, Universiti Kebangsaan Malaysia, 43600 Bangi, Selangor, Malaysia; ^2^Cluster of Integrative Medicine, Advanced Medical and Dental Institute, Universiti Sains Malaysia, Bertam, 13200 Kepala Batas, Penang, Malaysia; ^3^Department of Parasitology, Faculty of Medicine, University of Malaya, 50603 Kuala Lumpur, Malaysia; ^4^Faculty of Pharmacy, University of Technology MARA, 42300 Bandar Puncak Alam, Selangor, Malaysia

## Abstract

*Plasmodium falciparum* mitogen-activated protein (MAP) kinases, a family of enzymes central to signal transduction processes including inflammatory responses, are a promising target for antimalarial drug development. Our study shows for the first time that the* P. falciparum* specific MAP kinase 2 (PfMAP2) is colocalized in the nucleus of all of the asexual erythrocytic stages of* P. falciparum* and is particularly elevated in its phosphorylated form. It was also discovered that PfMAP2 is expressed in its highest quantity during the early trophozoite (ring form) stage and significantly reduced in the mature trophozoite and schizont stages. Although the phosphorylated form of the kinase is always more prevalent, its ratio relative to the nonphosphorylated form remained constant irrespective of the parasites' developmental stage. We have also shown that the TSH motif specifically renders PfMAP2 genetically divergent from the other plasmodial MAP kinase activation sites using Neighbour Joining analysis. Furthermore, TSH motif-specific designed antibody is crucial in determining the location of the expression of the PfMAP2 protein. However, by using immunoelectron microscopy, PPfMAP2 were detected ubiquitously in the parasitized erythrocytes. In summary, PfMAP2 may play a far more important role than previously thought and is a worthy candidate for research as an antimalarial.

## 1. Introduction

The spread of antimalarial resistance, in* Plasmodium falciparum* populations, is an important factor in our inability to effectively control this deadly cause of malaria. Consequently, there is an urgent need to identify novel targets for a new generation of antimalarials. MAP kinases have a potential role in the regulation of cell cycle machinery of the malaria parasite upstream of the signal transduction pathways involved in the control of* Plasmodium falciparum* proliferation. The* Plasmodium* parasite is unable to survive in the human red blood cell without protein kinases [1].* Plasmodium* kinases first reported in 1997 [2, 3] are a promising target for new antimalarials due to divergence in the properties and phylogenetic differences between* Plasmodium* and the host which leaves a space to exploit and inhibit specific enzymes that are crucial to* Plasmodium* [4–6].

In* P. falciparum*, two types of MAP kinases are known: PfMAP1 and PfMAP2 [7]. Initially, only PfMAP1 was thought to be expressed in both sexual and asexual stages of the malaria parasite. Further studies confirmed that PfMAP2 is also expressed in both stages, sexual and asexual. Both MAP kinases differ from a normal 3-cascade MAPK usually found in eukaryotes (MAPKKK-MAPKK-MAPK). Reverse genetic analysis showed that PfMAP2 plays an important role in* P. falciparum* survivability in the intraerythrocytic life cycle [7]. PfMAP2 is also known to play a compensatory role to PfMAP1 [7]. Although PfMAP1 does not play an essential role in schizogony and Gametocytogenesis its knockout harbours an increase in PfMAP2 protein suggesting PfMAP1 is necessary for asexual cycle survivability [3, 7]. PfMAP2 possesses an atypical activation site which is the TSH motif instead of the TXY motif conserved in MAPKs in other eukaryotes [3]. PfMAP2 is unique in that it cannot be confined to a specific MAPK family. Although both enzymes were originally grouped within the extracellular signal-regulated kinase 1, ERK1/ERK2 family of MAP kinases, a comprehensive phylogenetic analysis [4] of the entire complement of human protein kinase sequences indicates that PfMAP1 is clearly closer to the recently described ERK7/8 [8, 9]. The uniqueness of PfMAP2 is further strengthened by the finding that no members of the MAPKK family are found in the* Plasmodium* genome [8, 9].

Thus, PfMAP2 is seen as a potential drug target for further investigation in hemotherapeutics. However, very little is known about this protein and the function of PfMAP2 is poorly understood. This study was carried out to comprehend and enhance the understanding of PfMAP2 protein in order to convey it as a potential drug target.

## 2. Material and Methods

*(i) In Vitro Culture of P. falciparum*.* P. falciparum* 3D7 strain was cultured with some alterations [10, 11]. Percentage of* P. falciparum* used ranged between 8% and 10% parasitemia at 5% hematocrit.

*(ii) Synchronization of P. falciparum Culture*.* P. falciparum* culture was synchronized using 5% D-Sorbitol as described by Radfar et al. [11]. Briefly, the culture was pooled and centrifuged at 2000 ×g for 5 minutes at room temperature. Supernatant was discarded and 5% D-Sorbitol was added at a ratio of 1 : 1 (v/v) for 15 minutes followed by centrifugation (2000 ×g, 5 minutes, at room temperature). Pellets were washed with washing medium (RPMI1640, gentamycin, HEPES, and hypoxanthine) twice before being added into a new culture flask containing complete medium and freshly processed O+ type blood at 2% hematocrit.

*(iii) Extraction of P. falciparum Protein*. Synchronized cultures of* P. falciparum *were collected in a 15 mL tube. Subsequently, proteins from the parasites were extracted. Parasites were subsequently harvested by suspending the red blood cell (RBC) for 10 minutes at room temperature in a saponin lysis buffer. Samples were centrifuged at 1300 ×g for 10 minutes and RBC was continuously lysed with saponin twice. Parasites were then washed with phosphate buffered saline (PBS) and the pelleted parasites were subsequently frozen at −80°C.

Lysis buffer containing 1% Triton-X, 50 mM Tris HCl pH 8, protease inhibitor cocktail (Sigma USA), and phosphatase inhibitor cocktail were added to the parasite pellet at a concentration of 1 to 5 × 10^8^ parasites per millilitre. Samples were left on ice for 30 minutes and centrifuged at 13,000 ×g for 10 minutes. A total of 20 *μ*L supernatant containing the parasites were aliquoted for protein analysis using the Bradford assay [12]. The remaining supernatant was mixed equally with 2x-sample buffer (0.5 M Tris HCl pH 6.8, glycerol, 10% SDS, 0.05% 2-mercaptoethanol, and 0.5% bromophenol blue) boiled for 5 minutes and centrifuged at 250 ×g, 5 minutes.

*(iv) Western Blot*. Samples were subjected to SDS-PAGE [13] and blotted on a nitrocellulose membrane [14]. Immunoblotting was carried out using a rabbit-anti-PfMAP2 antibody and a rabbit anti-phosphorylated PfMAP2 antibody (PPfMAP2) at a dilution of 1 : 3000 overnight in +4°C. Both primary antibodies were prepared by Biogenes GmbH, Germany. Briefly, two New Zealand White rabbits were immunized with a synthetic peptide derived from PfMAP2 amino acid sequence retrieved from PlasmoDB database raised towards sequence CLKKQLTSHVVTR (residue 285–296). Terminal cysteine residue was added on the N-terminus in order to enable direct conjugation to the protein carrier. The purification step allows the antibody to differentiate between a phosphorylated and nonphosphorylated peptide. Rabbit-anti-phosphorylated-PfMAP2 serum is the phosphorylated form of the antibody which is the active form of the protein. The activation motif of PfMAP2 protein is reported to be TSH which is different from the common MAPK which is TXY [3]. The purified phosphorylated-specific antibody recognizes the phosphorylation site (-pT – pS). Membranes were washed using TBST solution three times followed by incubation for an hour on an orbital shaker (50 rpm) with anti-rabbit secondary antibody HRP-conjugated (1 : 15,000). Membranes were washed, incubated with Enhanced Chemiluminescent Western Blotting solution (ECL, Pierce) for 5 minutes prior to film exposure. Densitometry analyses of PfMAP2 and PPfMAP2 bands were performed using Bio-1D software [15].

*(v) Indirect Immunofluorescence Assay (IFA)*. The method used is based on Ramasamy et al. [16] and Dorin et al. [3] with some modifications. Blood smears of* P. falciparum-*infected red blood cells were prepared on slides at different stages of the parasite life cycle. Slides were then fixed with a mixture of methanol and acetone (1 : 1 v/v) for one minute. Control slides were prepared by incubating slides with 200 U of Lambda phosphatase (Calbiochem USA) for 30 minutes at 4°C. Next, 10% Fetal Bovine Serum (FBS) diluted in 1x PBS was used to block the slides at 37°C for 2 hours. Slides were then washed with 1x PBS for 5 minutes, 3 times followed by primary antibody incubation (PfMAP2 or phosphorylated PfMAP2 antibody at 1 : 100 dilutions) for 1 hour at 37°C. Subsequently, slides were washed at intervals of 5 minutes for 3 times. Next, slides were incubated in secondary antibody (conjugated FITC at 1 : 200 dilution) for 1 hour at 37°C and then were washed with PBS. Slides were then stained with 4′, 6-diamidino-2-phenylindole (DAPI) (2 *μ*g/mL) for 30 minutes at 37°C. After incubation, slides were washed with 0.05% phosphate buffered saline-Tween (PBST) for 2 times and once with 1x PBS. Slides were mounted with mounting medium and cover-slipped. Slides were observed using Olympus fluorescence microscope and images were analyzed using Image Analyzer.

*(vi) Statistical Analysis*. The effect of parasite stage of development and phosphorylation status on the relative intensities (expression) of PfMAP2 were analyzed using a repeated measures 2-way ANOVA, with a Bonferroni post test (Graph Pad Prism).

*(vii) Phylogenetic Analysis*.* Plasmodium* and human MAP kinase sequences were retrieved from PlasmoDB and GenBank, respectively. Similarity searches were done using the Basic Local Alignment Search Tool (BLAST). A total of 30 amino acids which contain the activation motives from each MAP kinases were used. The activation motif of* Plasmodium* MAP1 is TDY [3], TSH for* Plasmodium* MAP2 [3, 17], TEY for ERK [18], TGY for p38 [19], and TPY for JNK [20]. MEGA 4 software was used to construct the Neighbour Joining (NJ) phylogenetic tree with 1000 bootstrap replicates. NJ analyses were performed with distances calculated with the p-distance parameter [21–23].

*(viii) Bioinformatic Analysis of Localization of PfMAP2*. All computational studies were performed on a few bioinformatics tools able to predict the subcellular localization of PfMAP2 protein. PfMAP2 sequence was retrieved from PlasmoDB (http://www.plasmodb.org/). Subcellular localization of PfMAP2 protein was carried out using program CELLO [24, 25], BaCelLo [26], Loctree [27], PProwler [28], Euk-mPLoc 2.0 [29–32], and PredictProtein [33]. Nuclear localization signals, PredictNLS [33], and NetNES 1.1 [34] in PfMAP2 and analysis of PfMAP2 protein were done using Signal Peptide [35] and Secretome v 2.0 [36].

*(ix) Immunoelectron Microscopy (IEM) of Infected Erythrocyte*. Immunoelectron microscopies were performed as described by Bannister and Kent [37].* P. falciparum*-infected blood cultures were collected in a 15 mL tube and subsequently centrifuged (2000 ×g, 15 minutes). The pellet was suspended and fixed in McDowell Trump fixative prepared in 0.1 M phosphate buffer (pH 7.2) [37, 38]. Fixed specimens were washed for a few times, dehydrated, and embedded in London White Resin [39]. Ultrathin sections about 70 nm in thickness were collected by using 200–300-mesh nickel grids. The grids were incubated in blocking reagents for 30 minutes at room temperature and then incubated in primary antibody (rabbit-anti-PPfMAP2 antibody) for two hours at room temperature (dilutions of 1 : 10 were used). After repeated washing in TBS-Tween 20 buffer for 5 times, 5 minutes each, the grids were incubated in goat anti-rabbit IgG conjugated to 5 or 10 nm gold particles with dilution of 1 : 50. After washing step with the same buffer, the grids were stained with uranyl acetate for 2 minutes and examined with a transmission electron microscope H-9500.

## 3. Results and Discussion

**Figure 1:**
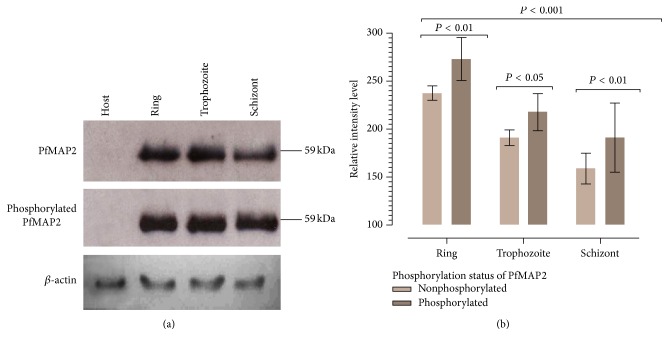
Expression levels of PfMAP2 at different stages of* P. falciparum*. (a) Expression pattern of PfMAP2 and phosphorylated PfMAP2 (PPfMAP2) within the developmental stages of* P. falciparum*. Stage-specific Western blot analyses were performed. H, host; R, ring stage; T, trophozoite stage; S, schizont stage. *β*-actin was used as a standard to allow semiquantitative comparison between samples over time. (b) Result shown is the average value of triplicate result (*n* = 3) +SE. A repeated measures 2-way ANOVA with Bonferroni post hoc analysis showed that the overall RI of (PfMAP and PPfMAP2) significantly decreased as the parasite developed from ring to schizont (*F* = 43.8, DF = 2,12 *P* < 0.0001), and that the RI of PPfMAP2 was always significantly higher than PfMAP2. However the ratio of PfMAP2 and PPfMAP2 did not significantly differ between stages of development; that is, there was no interaction effect (*F* = 0.28, DF = 2,12 *P* = 0.759 (not shown in the figure)). Immunoblotting was carried out using PfMAP2 peptide and PPfMAP2 peptide (1 : 3000).

Our studies showed that not only is PfMAP2 expressed in all stages of asexual* Plasmodium* development (Figure 1), it is also expressed specifically in the nucleus irrespective of MAPK activation suggesting a more profound role being played by PfMAP2 in contrast to PfMAP1.

We obtained a single band of 59 kDa in our Western blot using the PfMAP2 and PPfMAP2 antibodies (Figure 1(a)).  Figure 1(b) showed that the intensity of PfMAP2 protein is highest (2.3 × 10^5^) at the ring stage compared to the trophozoite (1.9 × 10^5^) or schizont (1.6 × 10^5^). The percentage of activation, phosphorylated protein, is about 15.73% more compared to the nonactive form of the protein during ring stage. In the trophozoite stage, the percentage of activation is slightly lower with total of 14.17%. During the schizont stage, although the amount of PfMAP2 protein is lowest compared to the rest, its phosphorylated intensity value is highest at 16.69% more than the nonphosphorylated PfMAP2. Our studies also showed a greater degree of PfMAP2 expression in the ring stage as opposed to the trophozoite and schizont stages suggesting that PfMAP2 is required for* Plasmodium* maturation since it is widely accepted that the ring stage undergoes the most prolific cell differentiation compared to the other asexual stages [4]. This is supported by microarray results [4] which showed higher Pfmap2 mRNA in ring stage as compared to later stages. A functional* map2* gene is required for* P. falciparum* asexual growth [7]. The results showed that expression of PfMAP2 protein is not gametocyte-specific. The presence of both PfMAP2 and phosphorylated PfMAP2 in all stages supports the idea that PfMAP2 plays a crucial role [40] in the* P. falciparum* asexual stage development of the asexual cycle especially during the phase of parasite growth and maturation.

We have also provided evidence showing that the blocking peptide binds completely to the PfMAP2 and PPfMAP2 antibodies as shown by the lack of signal on the Western blots (data not shown). This confirms that both the phosphorylated and nonphosphorylated form of antibodies generated using the TSH motif is highly specific. Therefore, the signals generated in the Western blots and IFA are likely indicative of the expression and localization of the PfMAP2 protein in the parasite. This is the first report on the localization of PfMAP2 in the nucleus of the parasite as contrary to the previous report of PfMAP2 being present in the cytoplasm of the parasite [7]. We explain this as our antibody centres around the TSH motif whereas the previously published expression of PfMAP2 was based on the construction of a knock-in which lacked the complete TSH motif [7]. Moreover, although the expression of PfMAP2 in our study is concentrated in the nucleus, there is a certain degree of expression in the cytoplasm in the areas closest to the nucleus and this is somewhat in agreement to what was observed in the previous report [7] that there exists an overlap in expression in the cytoplasm as well as the nucleus.

**Figure 2:**
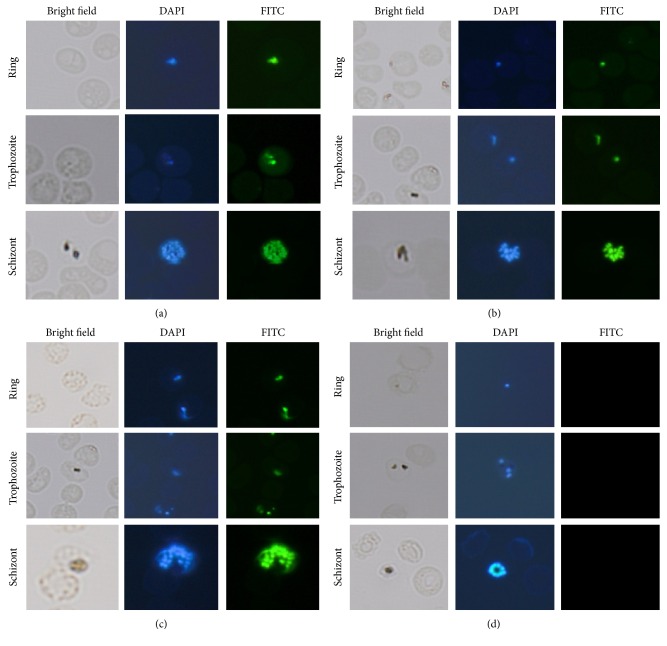
Subcellular distribution of PfMAP2 in* P. falciparum*. Immunofluorescence analysis of (a) PfMAP2 without phosphatase treatment localization in* P. falciparum*-infected red blood cells, (b) PfMAP2 with phosphatase treatment localization in* P. falciparum*-infected red blood cells, (c) PPfMAP2 without phosphatase treatment localization in* P. falciparum*-infected red blood cells, and (d) PPfMAP2 with phosphatase treatment localization in* P. falciparum*-infected red blood cells.* P. falciparum*-infected red blood cells were treated with DAPI (blue) and incubated with primary antibody PfMAP2 (1 : 100) and secondary antibody conjugated-FITC (green) (1 : 200).

Figure 2(a) shows the Indirect Immunofluorescent Analysis (IFA) using PfMAP2 antibody without phosphatase and Figure 2(b) shows PfMAP2 antibody with phosphatase treatment. While Figure 2(c) shows the IFA result with PPfMAP2 antibody without phosphatase treatment Figure 2(d) shows PPfMAP2 antibody with phosphatase treatment. All results barring the control experiment shown in Figure 2(d) showed that PfMAP2 and PPfMAP2 are localized in the parasite nucleus in all intraerythrocytic stages (ring, trophozoite, and schizont). We explain this in terms of the importance of subcellular localization playing a vital role in physiological functions, for example, in metabolic pathways, signal transduction cascades, and structure associated functions [41]. For a protein to function properly, translocation to the right compartments (intra/extracellular) occurs when the protein assumes a soluble form or is attached to a membrane [41]. MAP kinases activated in the nucleus will activate the mitogenic response and hence aid in parasite survivability. Several other proteins are reported to be translocated into the nucleus to play important roles in parasite survivability. Similar to PfMAP2,* Pf60* gene is located within the nucleus in all mature blood stages [42] and its possible function is in regulating homo- or heteromeric interactions in the parasite nucleus. Apart from* Pf60* gene, an unusual peroxiredoxin protein of* P. falciparum*, PfnPrx (earlier known as MCP1) is also localized to the nucleus of* P. falciparum.* PfnPrx is associated with the parasite chromatin suggesting its potentially essential role in the protection of nuclear DNA against oxidative stress which is similar to* Pf60* gene. Pfmrk is similar to mammalian CDK7 and is activated by PfMAT1. It is interesting to note that even though the nuclear localization signal (NLS) sequence is absent in the amino acid sequence of the parasite [43], both Pfmrk and PfMAT1 proteins are localized in the nucleus. Pfmrk activates downstream substrates; PfRFC-5 and PfMCM6, hence suggesting that Pfmrk is important in regulating DNA synthesis [43].

**Figure 3:**
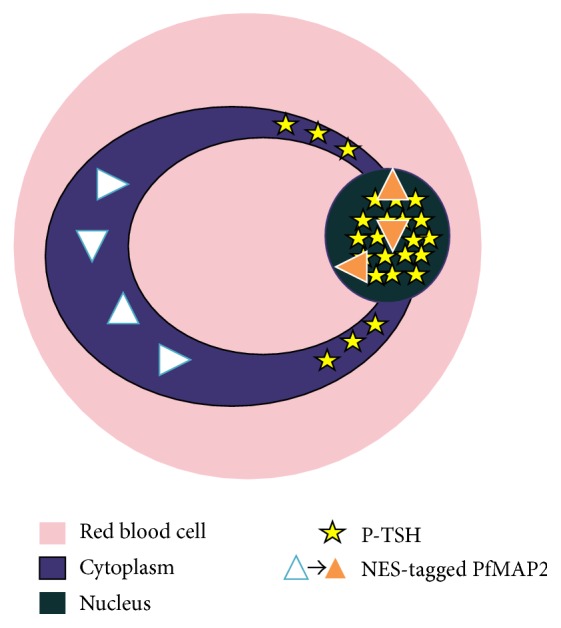
Schematic representation of the localization of phosphorylated PfMAP2. PPfMAP2 was shown to be in the nucleus. Expression of PPfMAP2 also extends into the cytosol in the areas surrounding the nucleus (P-TSH). NES-tagged PfMAP2 are thought to be transported from the cytosol to the nucleus. Upon phosphorylation, it will localize to the nucleus and activate downstream substrates.

The PfMAP2 sequence was analyzed using different bioinformatics prediction software as described in the methodology section and shown in Table 1. Four out of six bioinformatics software programs predicted PfMAP2 would be in the nucleus of the parasite. The NES sequence (nuclear export signal) is found to be present in the PfMAP2 sequence; hence the likelihood of it aiding nuclear import-export mechanism in the parasite is high. Interestingly, NES is a lysine residue which potentially interacts with the receptors that export out protein from the cell's nucleus [44] (Figure 3). Upon further investigation, PfMAP2 does not contain a nuclear localization signal (NLS). NLS is a signal or sequence in a protein which transports protein into the cell nucleus by nuclear transport. The function of NLS is known to be in opposition to NES [42, 44, 45].

Furthermore, removal of the phosphorylation activity of the parasite by the phosphatase treatment further supports the specificity of the PPfMAP2 antibody which we designed focussing on the TSH motif due to the complete lack of signal as shown in Figure 2(d). MAPK is activated when it is phosphorylated on both threonine and tyrosine [46]. However, in* P. falciparum*, PfMAP2 is shown to have atypical property of MAPK as its activation motif is TSH as compared to typical MAPK which is TXY [2, 3]. This divergence of the plasmodial MAPK from the human protein kinase makes them attractive as a potential and promising drug target [5, 47, 48]. The human phosphorylated mitogen-activated extracellular signal regulated protein kinase (MAPK/ERK-P), phosphorylated protein kinase of 38 kDa (p38-P), and phosphorylated stress-activated protein kinase (SAPK/JNK-P) expression have been examined in Alzheimer's disease (AD), Pick's disease (PiD), progressive supranuclear palsy (PSP), and corticobasal degeneration (CBD) [49]. Inhibition of ERK activation reduced* P. falciparum* oocyst load and infection prevalence in* Anopheles stephensi* as well as enhancing TGF-b1-mediated control of* P. falciparum* development [50]. PfMAP2 is reported to be related to SNF-1 family [4]. SNF-1 is a subfamily of protein kinase group which plays a crucial role in regulating lipid metabolism and cell stress response [51]. Activation of PfMAP2 protein in all the stages supports the parasite metabolism by activating downstream substrates which might be diverted compared to mammalian MAP kinases.

**Figure 4:**
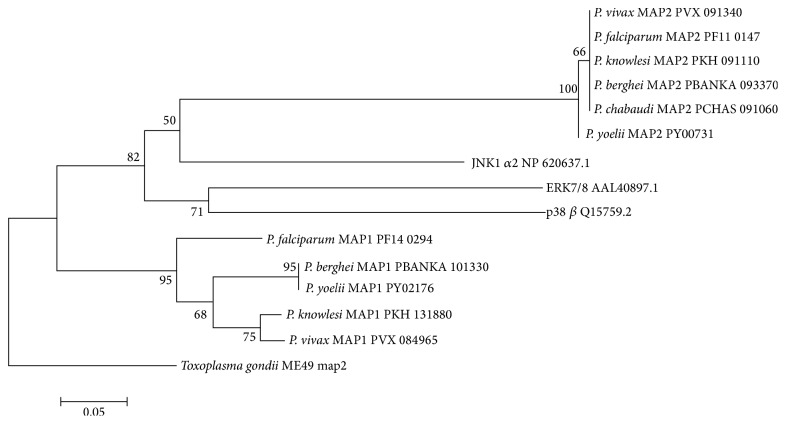
Phylogenetic analysis of PfMAP2 protein. Phylogenetic analysis using Neighbour joining (NJ) tree displaying the genetic relationships among MAP kinases in* Plasmodium* species. Bootstrap support of more than 65% and distance is indicated.* Plasmodium* MAP kinases sequences were retrieved from PlasmoDB and* Homo sapiens* MAPK sequences representing ERK 7/8, JNK, and p38 were retrieved from NCBI.

We have also shown that the TSH motif of PfMAP2 sets it apart from PfMAP1 as our Neighbour Joining analysis with 1000 bootstrap replicates suggests that it is less related to both the human MAP kinases and the TXY motif of plasmodial MAP1 as shown in Figure 4. This is in agreement with the extensive divergence of the kinome of* P. falciparum* which showed that PfMAP2 is located in a basal position relative to other MAP kinases whereas PfMAP1 is clearly associated with the human MAP kinase, ERK8 [4]. The divergence of PfMAP2 from PfMAP1 as shown in Figure 4 using only the activation sites of these proteins suggests that the TSH motif is not only highly unique but it is also evolutionarily divergent from the other MAP kinases inclusive of plasmodial MAP1 and human MAP kinases.

The data shown do not allow us to draw any conclusion regarding detailed function of PfMAP2 during the intraerythrocytic cycle. However, the additional information on PfMAP2 protein could possibly aid in finding downstream substrates and hence predict PfMAP2 pathway that is involved in its protective mechanism similar to JNK/p38 and also assist in parasite proliferation which is similar to ERK function [52–54].

**Figure 5:**
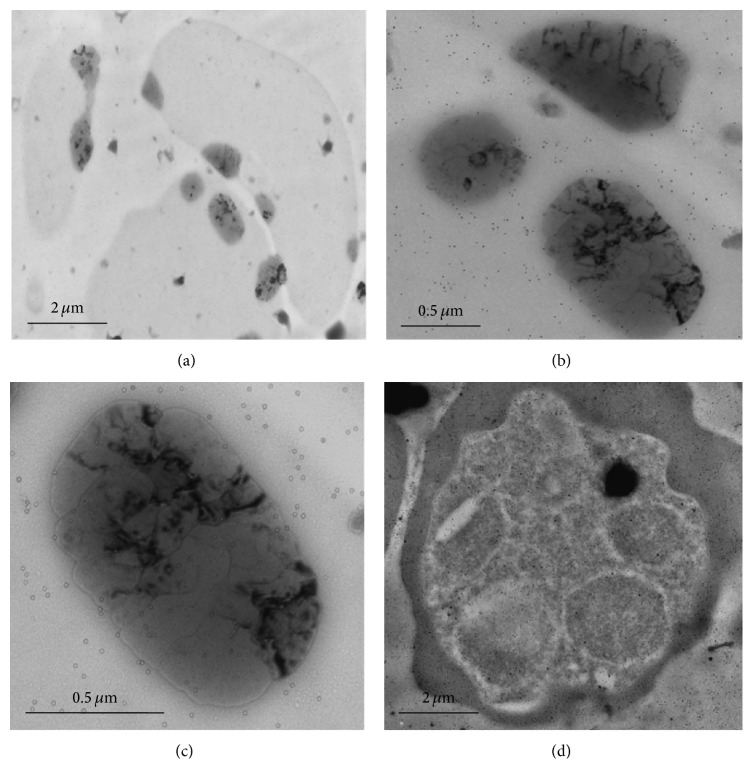
Localization of PfMAP2 by immunoelectron microscopy. Ultrathin sections of* P. falciparum* parasites at intraerythrocyte stage were labelled with rabbit anti-PPfMAP2 antibody followed with anti-rabbit secondary antibody conjugated with 15 nm colloidal gold particle. Ultrathin sections of infected erythrocyte were observed under 7 000x (a), 25 000x (b), and 40 000x (c) magnification. Similarly PPfMAP2 staining in the schizont was observed under 8 000x magnification (d).

Immunogold labelling together with the use of transmission electron microscopy would aid in a more precise localization of the activated protein. Therefore, to further confirm the localization of PfMAP2, ultrathin sections of parasitized red blood cell were probed with anti-PPfMAP2 antibodies. At lower magnification, PPfMAP2 staining was detected ubiquitously. When the parasitized erythrocytes were observed at higher magnification, the immunogold was detected in various vesicular structures including nucleus (Figure 5). Since we do not have a specific PPfMAP2 antibody which is nucleus-specific, we have come to understand that the expression of PfMAP2 is regionalised in various organelles of the* Plasmodium* parasite. Here, we suggest that since PfMAP2 have a functional role during the pathological development of the parasite [4], our data renders that further detailed investigation should be done on this protein. Further studies on the placement of PfMAP2 in the nuclear architecture will aid in understanding on the gene regulation involved in its function and the role of nuclear structure in MAP kinase antigen variation.

## 4. Conclusion

The finding that the PfMAP2 protein is highly localized in the parasite's nucleus at all intraerythrocytic stages and its expression is significantly dependent on the stage of* P. falciparum* asexual maturation further supports future potential manipulation of PfMAP2 as a protein kinase inhibitor to halt the spread of malaria.

## Figures and Tables

**Table 1:** PfMAP2 subcellular localization prediction through 6 available servers for eukaryotic proteins.

Bioinformatics tool	Subcellular localization
CELLO	Nucleus
BaCELLO	Cytoplasm-nucleus-cytoplasm
LocTree	Cytoplasm
PProwler	Other than mitochondria peroxisome, secretory pathway
Euk-mPLoc 2.0	Cytoplasm, nucleus
PredictProtein	Nuclear
